# Toward Sustainable Crop Production in a Changing Climate: Microbiomes, Pathogens, Biostimulants and Biocontrol Innovations

**DOI:** 10.1111/1751-7915.70442

**Published:** 2026-09-21

**Authors:** Ashleigh Holmes, Miguel A. Matilla

**Affiliations:** ^1^ Department of Cell and Molecular Sciences James Hutton Institute Dundee Scotland UK; ^2^ Department of Biotechnology and Environmental Protection Estación Experimental del Zaidín, Consejo Superior de Investigaciones Científicas Granada Spain

## Abstract

Climate change is recognised as one of the most pressing global challenges, with profound consequences for plant health, agricultural productivity, and food security worldwide. In this opinion article, we discuss recent advances in how climate change affects plant–microbe interactions, soil antibiotic resistance, and plant‐associated microbiomes, including shifts in the distribution of plant pathogens. We also examine the influence of land‐use practices, including integrated agricultural management, on the physicochemical and microbiological properties of soils under changing climatic conditions. In addition, we critically discuss emerging biological and biotechnological strategies aimed at enhancing plant health and resilience under climate change. These approaches include the rational design of synthetic microbial communities (SynComs), bacteriophage therapy, mycovirus‐based biocontrol, antibiotic‐producing microbial biocontrol agents, (nano‐enabled) microbiome engineering, and extracellular vesicle‐mediated RNA delivery systems. Challenges involved in translating laboratory‐based discoveries into effective field applications are also discussed. Collectively, current advances highlight the growing potential of microbial biotechnology to reduce reliance on chemical pesticides and fertilisers, while supporting sustainable and integrated crop management under climate‐change scenarios.

## Graphical abstract

Overview of the impacts of climate change on plant–microbe interactions and the plant microbiome, as well as emerging microbial biotechnological strategies for sustainable crop protection.

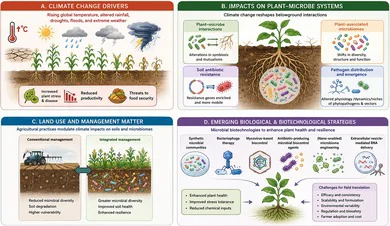

## 1 Background

Climate change is recognised as a major driver of environmental and societal disruption at the global scale. Its effects are manifested through a sustained rise in average temperatures, accompanied by more frequent and intense heatwaves, as well as marked shifts in precipitation patterns that lead to prolonged droughts and more frequent flooding events. This growing climatic instability exerts significant pressure on natural systems and human societies, positioning climate change as one of the most pressing global challenges. Indeed, climate change is projected to have profound impacts on global agricultural production. Current estimates suggest that each 1°C increase in global temperature could reduce annual global food production by approximately 5 × 10^14^ kcal, equivalent to a loss exceeding 4% of the recommended daily caloric intake worldwide (Hultgren et al. 2025). Compounding this challenge, crop losses caused by phytopathogens are estimated to reduce global harvests by up to 40% annually, with economic costs of approximately US$300 billion (Savary et al. 2019; Holtappels et al. 2021; Singh et al. 2023). These impacts will be aggravated by climate change, which is expected to alter the physiology and dynamics of phytopathogens and their vectors, expand their ecological niches, weaken plant immune responses, and reduce the diversity and core functions of beneficial plant microbiomes (Xiong, Ge, et al. 2026), thereby compromising plant health and productivity; ultimately threatening global food security. Beyond its effects on agricultural systems, climate change also has important implications for public health, as increasing evidence links it to the rise of antibiotic resistance in human pathogens (Kalanxhi and Laxminarayan 2026). In this opinion article, we present and critically evaluate recent evidence on the impacts of climate change on plant–microbe interactions, the emergence of antibiotic resistance in soils, and climate‐driven changes in plant‐associated microbiomes. We further discuss emerging (bio)control strategies to mitigate the growing threat of biotic and abiotic stresses under a changing climate.

## 2 Climate‐Driven Dynamics of Soil Microbiota and Plant–Microbe Interactions

Soils and their associated microbiota are highly vulnerable to the impacts of climate change. A recent large‐scale metagenomics study revealed that the global distribution of phytopathogens is strongly shaped by microbiological and environmental factors, with warm ecosystems and intensively managed agricultural soils favouring phytopathogen prevalence, whereas colder climates, higher soil organic carbon content, and greater microbial diversity were associated with lower abundance (Gao et al. 2026). Although 32 globally dominant phytopathogens, including species from the genera *Agrobacterium*, *Clavibacter*, *Ralstonia*, *Burkholderia*, *Pseudomonas*, and *Xanthomonas*, were identified across diverse environments, their enrichment in genes associated with plant interaction processes, such as signalling, sugar transport, energy production, and carbohydrate metabolism compared with non‐dominant bacteria taxa supports a consistent adaptation to plant host‐associated lifestyle. Notably, the results reinforce the concept that the capacity of soils to suppress plant diseases is not an intrinsic soil property but driven by key microbial constituents, including the presence of arbuscular mycorrhizal fungi and members of the phyla Actinomycetota and Bacillota, as well as to their genetic potential to synthesise bioactive secondary metabolites (Gao et al. 2026), which play an important role in protection against phytopathogens (Roca and Matilla 2023; Ding et al. 2025; Parveen et al. 2026). Predictive models further suggest that climate change may promote the global spread of important bacterial phytopathogens (Gao et al. 2026), which is crucial for surveillance and deploying effective (bio)control strategies. Notably, these patterns are not restricted to phytopathogens, as the same authors have reported similar trends for the distribution of human pathogens across global soils (Xiong, Delgado‐Baquerizo, et al. 2026). However, despite broad spatial and climatic coverage across different vegetation types and both agricultural and natural ecosystems, these models should be interpreted with caution, as they rely on correlative metagenomic signatures, while sampling biases and the simplification of highly complex and dynamic environments may affect interpretation, limiting, for example, the equal extrapolation of results across pathogens or microbiome components.

Currently, 2.1 billion people lack access to safely managed drinking water (United Nations 2026). The extent of global terrestrial land affected by multiyear drought events is increasing at an alarming rate (Chen, Brun, et al. 2025), posing a growing threat to ecosystems and human populations in a changing climate. By 2050, an estimated 5 billion people are expected to live in water‐scarce regions, as agricultural water demand doubles and freshwater availability may decline by up to 50% due to climate change (Gupta et al. 2020). In this context, drought has been investigated as the selective pressure shaping the emergence of resistance to antibiotics in soils across different land uses and geographic regions (Shan et al. 2026). This effect was attributed to reduced soil water content, which may increase the concentrations of naturally occurring antibiotics in the soil matrix, independent to the relative expression of antibiotic biosynthetic genes. Under more severe drought conditions, this effect was associated with a higher abundance of microbial genes involved in both antibiotic biosynthesis and resistance, driving shifts in the functional composition of soil microbial communities towards more resistant phenotypes (Shan et al. 2026). Although projections from this study suggest that climate change is likely to drive an increase in antibiotic resistance across all inhabited continents, further studies are required to assess the effect of drought in soils with different chemical compositions and across a broader range of climatic and geographical conditions. Such studies are particularly important given the chemical and biological complexity and heterogeneity of soil systems, which can influence the production, activity and stability of antibiotics. Against this backdrop of climate‐driven drought stress and microbiome restructuring, agricultural management practices emerge as critical modulators of plant resilience. Thus, comparisons between conventional (i.e., high agrochemical inputs, intensive soil disturbance, and simplified rotations) and integrated (i.e., reduced tillage, use of biostimulants, and plant‐derived extracts) agricultural management under recurrent drought over decades support that integrated practices can improve key soil properties, including phosphorus availability, water content, and C:N ratios, while also reshaping microbial community composition towards taxa with stress‐adaptive traits. These changes include an enrichment of spore‐forming bacteria from the *Bacillaceae* family, which alleviate plant drought stress under greenhouse conditions (Ruiz‐Muñoz et al. 2026). Although evidence from controlled experimental systems requires further validation under more realistic and environmentally variable field conditions before broad generalisation, these findings support the ‘cry for help’ hypothesis, whereby plants recruit beneficial microbes under biotic and abiotic stresses to enhance their resilience (Roca et al. 2024; Liu 2025). Likewise, the markedly reduced diversity of arbuscular mycorrhizal fungi in cultivated soils compared with uncultivated soils, together with its strong and differential modulation by climatic factors such as temperature and precipitation (Stewart et al. 2026), highlights the need to integrate land‐use practices and climate effects when designing conservation and sustainable agriculture strategies.

The use of synthetic microbial communities (SynComs) is emerging as a promising strategy to help plants cope with climate change–associated stresses, including salinity, heat, and nutrient limitations (Schmitz et al. 2022; Portal‐Gonzalez et al. 2025; Staiano et al. 2025; Zhu et al. 2025; Adhikary et al. 2026), as well as other stressors such as metals (Ma et al. 2024) and nanoplastic phytotoxicity (Xu, Liu, et al. 2026). In this context, the rational design of SynComs as a sustainable approach to mitigate drought stress has recently been explored by using desert plants as a source of drought‐adapted microbiomes (Hao et al. 2026). SynComs comprising up to 15 functionally and taxonomically distinct drought‐tolerance strains effectively promoted plant growth in major crops under drought stress more effectively than individual isolates in both sterile and non‐sterilised field soils, supporting the benefits of functionally complementary microbial consortia. Notably, their application under drought conditions induced compositional and functional shifts in the rhisosphere microbial communities and elicited changes in plant physiological and transcriptional responses, including the activation of signalling, antioxidant defence, and osmotic adjustment pathways (Hao et al. 2026). However, despite the encouraging results from laboratory‐scale studies, important questions remain regarding the dynamic interactions and functional compatibility among SynCom members, the long‐term stability and performance of SynComs under complex field conditions, integration with native microbial communities, and ecological impacts following repeated application (Xu, Shrestha, et al. 2026), particularly under the increasingly variable conditions imposed by climate change. Addressing these challenges will be essential before SynCom‐based technologies can be broadly and cost‐effectively implemented in sustainable agriculture. Rapid advances in multi‐omics technologies, high‐throughput interaction profiling, and computational approaches will provide a solid foundation for the custom design of SynComs with desired functional traits.

Soil salinisation affects more than 800 million hectares of land, markedly reducing agricultural productivity, with climate change further exacerbating its extent and intensity (Tang et al. 2024). Salinity stress has profound implications for the functionality of plant‐associated bacteria, including rhizobia. In this scenario, comparative genomic and metabolic analyses of more than 40 *Sinorhizobium meliloti* strains revealed the multifactorial nature of salt tolerance in this species, involving quorum sensing, exopolysaccharide and lipopolysaccharide biosynthesis, peptidoglycan transport, and carbohydrate metabolism, with salt‐tolerant strains exhibiting broader carbon source utilization capacities (Bellabarba et al. 2026). This highlights the need for additional studies to assess whether these genomic and metabolic signatures accurately predict strain performance under field conditions, where salinity is typically accompanied by multiple interacting abiotic stresses. Indeed, other climate‐related stressors such as soil warming further modulate plant–soil–microbe interactions. For example, warming has been shown to alter root‐derived soil carbon dynamics by reducing microbially driven carbon loss under elevated temperatures though changes in microbial biomass, soil microbial community composition, and functional potential (Chari et al. 2026). Collectively, these findings emphasize that future climate‐resilient microbial inoculants should be developed and evaluated under environmentally realistic conditions that account for interacting biotic and abiotic factors shaping microbial performance and plant responses.

## 3 Advances in the (Bio)Control of Plant Diseases Under Climate Change

Climate change will encourage the emergence of new diseases and the spread of existing pathogens into new regions, as discussed earlier (Singh et al. 2023; Xiong, Ge, et al. 2026). Unlike many synthetic pesticides, biocontrol agents do not leave persistent residues in soil, water, or food, and have less dependence on the petrochemical industry, resulting in a lower energy footprint. Biocontrol products are often highly specific and do not (generally) disrupt beneficial soil microbiota, thereby preserving soil health, which is essential for resilience to climate‐related stresses such as drought or salinity. The application of biological agents can be tailored and adapted to local conditions, providing a more targeted approach compared to traditional pesticide treatments. Reducing agricultural inputs is essential for meeting emission reduction policies and supporting sustainable agricultural systems (Hulot and Hiller 2021).

### 3.1 Direct Biocontrol Targeting of Phytopathogens

Understanding the specific mechanisms of the biological activity of biocontrol agents or bioinoculants is critical for their precise and effective application. Accordingly, the application of bacteriophage, including phage cocktails, for controlling bacterial plant diseases has been well described and evidenced to act synergistically with other treatments (Czajkowski et al. 2025). In addition, there is also emerging exploration into the application of mycoviruses, specifically hypovirulence‐associated viruses, for fungal pathogen control (Bi et al. 2025). The biocontrol activities of mycoviruses are more nuanced than phytopathogen host cell lysis imparted by bacteriophages, where non‐self‐responses can be silenced through co‐treatment with proline or horizontal spread may be promoted through zinc‐induced programmed cell death pathways (Bi et al. 2025).

Soil microbes are particularly valuable to bioprospecting for metabolites with biological activities of interest. A literature review covering the period 2018–2025 identified more than 320 natural products isolated from diverse soil‐associated microbial taxa, including both bacterial and fungal species that commonly interact with plant hosts (Rustamova et al. 2026). In this context, recent work on biocontrol strategies employed a comparative metabolomic and genomic approach to profile a collection of approximately 60 actinomycete strains exhibiting a broad spectrum of inhibitory activities against the phytopathogenic oomycete *Phytophthora infestans* , ultimately linking specific bioactive metabolites with disturbances in mycelial growth and zoospore germination (Abdelrahman et al. 2025). Similarly, screening of mycoparasitic species isolated from sclerotia enabled the isolation of *Paraphaeosphaeria minitans* as a promising biocontrol agent against *Sclerotinia sclerotiorum*. This bioactive isolate produces diverse metabolites, including the antifungal compounds linoleic acid and butyl octyl phthalate (Ruppavalli et al. 2026). Notably, the successful translation of these in vitro findings to field conditions demonstrated that *P. minitans* reduced cabbage head rot incidence to comparable levels of plants receiving fungicide treatments (Ruppavalli et al. 2026), emphasising its value as a sustainable biocontrol alternative to synthetic fungicides.

Microbe engineering to enhance the production of bioactive metabolites which have known antimicrobial properties to combat plant disease needs to be carefully controlled and potential effects on the wider ecology should be thoroughly considered. A recent *Microbial Biotechnology* publication investigated the engineering of a plant‐associated *Bacillus subtilis* strain to overproduce the antimicrobial lipopeptide surfactin as an approach to increase biocontrol potential against *Fusarium* banana wilt (Chen, Liu, et al. 2025). However, surfactin overproduction caused pleiotropic effects on the bacterium, affecting motility and biofilm formation, which ultimately reduced plant colonisation and biocontrol efficacy when applied to the banana plants. Notably, alterations to the surfactin production also influenced the banana rhizosphere community structures. A moderate increase of surfactin production promoted kin‐selective recruitment but hyperproduction reduced microbial community diversity and disease suppression (Chen, Liu, et al. 2025); supporting that the application of biocontrol agents should consider not only pathogen suppression efficacy, but also dosage‐dependent ecological effects.

### 3.2 Alternative Approaches for Biocontrol

Fundamental research into understanding the virulence mechanisms of phytopathogens under the context of climate change can lead to the identification of novel intervention strategies. For example, studying the biology of oomycetes, deciphering their genomes, and exploring their pathogenicity mechanisms have uncovered novelties and vulnerabilities that open new avenues for tailor‐made strategies for disease control (Wang et al. 2026). One such avenue is the exploitation of extracellular vesicles (EVs), which are employed by *Phytophthora infestans* for the delivery of effectors (Breen et al. 2025), while plants also produce EVs as part of their host defence responses that can be targeted by oomycete phytopathogens through the secretion of specific lipases (Xu, Kong, et al. 2026). Notably, EVs can be developed as delivery systems to overcome nucleic acid degradation challenges associated with RNA‐based antifungals approaches, such as spray‐induced gene silencing (Niño‐Sánchez et al. 2026). Engineered plant‐beneficial *B. subtilis* and *Pseudomonas putida* strains to produce EVs encapsulating antifungal double‐stranded RNAs (dsRNA) targeting *Botrytis cinerea* and *Verticillium dahliae* resulted in EVs transport into the fungal cells. Treatment with either purified EVs or the engineered bacterial strains significantly reduced infections caused by these phytopathogenic fungi in both model and crop plant systems (Niño‐Sánchez et al. 2026). Although this cross‐kingdom delivery offers a promising approach for future sustainable crop protection technologies, the diversity of cargos transported by phytobacterial EVs also implies a broad range of ecological functions within plant‐associated niches, including nutrient acquisition, antimicrobial activity, plant cell wall degradation, modulation of plant immunity, horizontal gene transfer, and intra‐ and inter‐kingdom communication (Zannis‐Peyrot et al. 2025). Consequently, their potential impacts on microbial communities and the broader plant ecosystem should be carefully evaluated to ensure their safe and responsible deployment in agricultural systems.

The integration of nanotechnology with microbiome engineering is emerging as an innovative microbial‐based approach to combat vascular phytopathogens, with the potential to provide more targeted and efficient alternatives to conventional fertilisers and pesticides (Ahmed et al. 2026). In this regard, different nanomaterials, such as nanochitosan biostimulants, can modulate the composition and activities of rhizosphere microbiomes, thereby improving plant health and resilience (Ahmed et al. 2023). These technologies could also be used to bolster defences against pathogen stress through the improved delivery of important nutrients or Syncoms (Ahmed et al. 2026).

## 4 Concluding Remarks

Over recent decades, extreme climate events (e.g., floods, droughts, heatwaves) have resulted in estimated global economic losses of around US$4.3 trillion, with agriculture among the hardest‐hit sectors. Major staple crops such as maize, wheat, potato, and rice have been particularly affected, with extreme events accounting for up to 27% of the observed variability in production yields (Xiong, Ge, et al. 2026).

**FIGURE 1:**
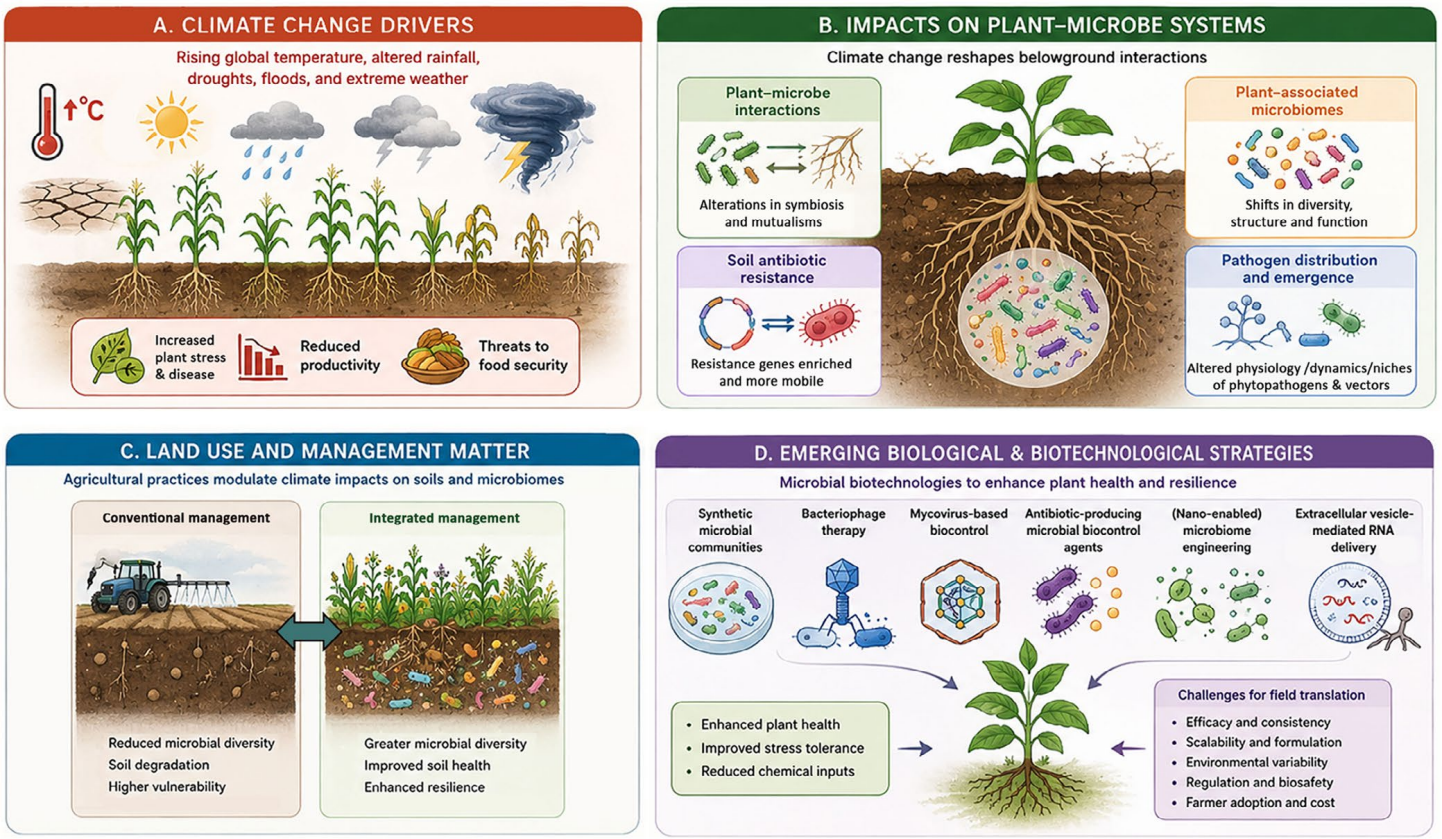
Overview of the impacts of climate change on plant–microbe interactions and emerging microbial biotechnology strategies for sustainable crop growth and protection. Climate change drivers (A) reshape belowground interactions, including plant–microbe interactions and plant‐associated microbiomes, and alter pathogen distribution and soil antibiotic resistance (B). Agricultural management practices modulate the effects of climate change on soils and microbiomes (C), while emerging biological and biotechnological approaches offer new opportunities to enhance plant health and resilience under changing climatic conditions (D). The figure was created with the assistance of ChatGPT‐4 (OpenAI; accessed June 2026) to generate an initial draft. All content was reviewed, edited, and validated for accuracy by the authors.

Numerous studies have identified soluble and volatile bioactive compounds, as well as biological agents, through in vitro assays. However, a major knowledge gap remains for the translation of these agents to real‐world field settings, where a multitude of environmental and ecological factors can impact efficacy (Belt et al. 2026). As microbiome research continues to expand in the context of understanding biocontrol action, there is considerable scope for future development of technologies to promote beneficial plant‐associated microbiomes to improve plant vigour against both biotic and abiotic stresses caused by climate change (Figure 1). Such technologies include plant mycoviral vaccines, where systemic infection provides broad fungal resistance (Bi et al. 2025); nano delivery of Syncoms or gene‐editing tools for optimised microbiome recruitment (Ahmed et al. 2026); and microbial modulation of crop architecture (Chen et al. 2026) (Figure 1).

The current market size of biofertiliser is estimated between US$2.9 billion and US$3.72 billion and is projected to grow at an annual rate exceeding 10% (Singh et al. 2025). At present, the online database Bio4safe (https://bio4safe.eu/) lists more than 280 microbial‐based products available as biostimulants to enhance plant resilience to climate change–associated stresses (e.g., drought, heat, salinity, water‐use efficiency, nutrient use efficiency). In parallel, the Centre for Agriculture and Bioscience International (CABI) offers a portal (https://bioprotectionportal.com/find‐products/) where users can explore the database of nationally registered biological crop protection products. Despite the enormous potential of live bioinoculants, including engineered microbial inoculants and RNA‐based delivery systems, regulatory bottlenecks and the lack of internationally harmonised regulatory frameworks remain major barriers to their widespread implementation. For example, in the European Union, current legislation is poorly adapted to living products, with microbial biostimulants and plant protection products regulated under separate frameworks (Hendriksen 2022; Köhl 2025; van der Berg et al. 2025). Likewise, the regulatory landscape for RNA interference (RNAi)‐based crop protection differs considerably among countries (Dietz‐Pfeilstetter et al. 2021; Sergi et al. 2026). These regulatory discrepancies, together with unresolved issues related to environmental risk assessment and biosafety, continue to hinder the efficient translation of these innovative technologies into sustainable agricultural practice. Nevertheless, continued advances in plant and microbial biotechnology, coupled with more coherent regulatory frameworks, will provide important opportunities to support the growing global demand for crop production whilst driving integrated crop management practices aligned with the United Nations Sustainable Development Goals.

From a technology readiness perspective, the approaches discussed in this opinion article span different levels of maturity, scalability, and field validation. Microbial inoculants are already broadly used and represent the most advanced approaches. Although phage‐based applications and SynComs are also being implemented, they are still evolving, with increasing mechanistic insight and advances in formulation, yet their validation under diverse and complex field conditions remains limited. By contrast, EVs, RNA delivery systems, and nano‐enabled microbiomes remain early‐stage technologies, with mostly proof‐of‐concept evidence and requiring further progress in scalability, regulation, and field testing before broader agricultural application. Importantly, these approaches are not mutually exclusive and may be integrated in complementary strategies, collectively representing significant advances towards more resilient and climate‐adaptive agricultural systems. Given the differences in stability, applicability, and cost competitiveness among these technologies, their broad commercial adoption in sustainable agriculture is likely to face important initial challenges. However, supportive policy frameworks and governmental initiatives will play a key role in accelerating their implementation. For example, the European Green Deal aims to reduce the use of chemical pesticides in Europe by 50% by 2030, underscoring the need for the continued development and widespread adoption of novel interventions.

## Author Contributions

**Miguel A. Matilla:** conceptualization, funding acquisition, writing – original draft, visualization, writing – review and editing, project administration, resources. **Ashleigh Holmes:** conceptualization, funding acquisition, writing – original draft, writing – review and editing, project administration, visualization, resources.

## Funding

This work was supported by Scottish Government Environment, Natural Resources and Agriculture Research Program, JHI‐A1‐1, JHI‐A1‐2, JHI‐D3‐1. Spanish Ministry for Science, Innovation and Universities, PID2023‐146281NB‐I00.

## Conflicts of Interest

The authors declare no conflicts of interest.

## Data Availability

Data sharing not applicable to this article as no datasets were generated or analysed during the current study.
